# Alpha-Enolase Is Upregulated on the Cell Surface and Responds to Plasminogen Activation in Mice Expressing a ∆133p53α Mimic

**DOI:** 10.1371/journal.pone.0116270

**Published:** 2015-02-02

**Authors:** Sonal Sawhney, Kylie Hood, Alisha Shaw, Antony W. Braithwaite, Richard Stubbs, Noelyn A. Hung, Janice A. Royds, Tania L. Slatter

**Affiliations:** 1 Wakefield Biomedical Research Unit, University of Otago, Wellington, New Zealand; 2 Department of Pathology, Dunedin School of Medicine, University of Otago, Dunedin, New Zealand; 3 Children’s Medical Research Institute, University of Sydney, Westmead, Australia; Rush University Medical Center, UNITED STATES

## Abstract

The p53 protein is a master regulator of the stress response. It acts as a tumor suppressor by inducing transcriptional activation of p53 target genes, with roles in apoptosis, cell cycle arrest and metabolism. The discovery of at least 12 isoforms of p53, some of which have tumor-promoting properties, has opened new avenues of research. Our previous work studied tumor phenotypes in four mouse models with different p53 backgrounds: wild-type p53, p53 null, mutant p53 lacking the proline domain (mΔpro), and a mimic for the human Δ133p53α p53 isoform (Δ122p53). To identify the major proteins affected by p53 function early in the response to DNA damage, the current study investigated the entire proteome of bone marrow, thymus, and lung in the four p53 models. Protein extracts from untreated controls and those treated with amsacrine were analyzed using two-dimensional fluorescence difference gel electrophoresis. In the bone marrow, reactive proteins were universally decreased by wild-type p53, including α-enolase. Further analysis of α-enolase in the p53 models revealed that it was instead increased in Δ122p53 hematopoietic and tumor cell cytosol and on the cell surface. Alpha-enolase on the surface of Δ122p53 cells acted as a plasminogen receptor, with tumor necrosis factor alpha induced upon plasminogen stimulation. Taken together, these data identified new proteins associated with p53 function. One of these proteins, α-enolase, is regulated differently by wild-type p53 and Δ122p53 cells, with reduced abundance as part of a wild-type p53 response and increased abundance with Δ122p53 function. Increased cell surface α-enolase on Δ122p53 cells provides a possible explanation for the model’s pro-inflammatory features and suggests that p53 isoforms may direct an inflammatory response by increasing the amount of α-enolase on the cell surface.

## Introduction

The p53 tumor suppressor is an intrinsic part of the cellular stress response [1]. Functions attributed to p53 continue to be discovered, including roles in determining cell fate and in energy metabolism, cell differentiation, embryo implantation, angiogenesis, migration, and inflammation (reviewed in [2–6]). To add to the complexity of understanding p53 function, many modifications of p53 exist, including 12 isoforms produced by the use of alternative promoters, splicing, and alternative sites of translation [7–12]. The Δ 133p53α isoform lacks the N-terminal 133 amino acids due to an alternative promoter in intron four; it is expressed in many normal tissues and aberrantly expressed in multiple tumors, including those of the breast, colon, and bile duct [8,13,14]. Tumor-promoting properties attributed to Δ 133p53α include angiogenic, proliferative, and inflammatory functions [7,15–18].

Many studies aimed at further refining p53 and p53 isoform function have focused on changes in gene expression. In this study we used a proteomic-based approach to discover new proteins associated with the wild-type p53 DNA damage response and p53 isoform function. The bone marrow, thymus, and lung proteomes from four different p53 murine models treated with or without a DNA damaging agent were compared using two-dimensional fluorescence difference gel electrophoresis (2D-DIGE). The p53 genotypes included wild-type mice (p53+) to investigate the normal p53 response, p53 null mice (p53-) as a control for the absence of p53 function, mice without the proline domain in p53 (mΔpro) previously shown to have an attenuated p53 response to DNA damage [19,20] and Δ122p53 mice [21,22]. Mice expressing the Δ122p53 protein that mimics the human Δ133p53α isoform can be used to study oncogenic properties, including pro-proliferative and pro-inflammatory functions and abnormal hematopoietic cell distribution in the lung and liver. Δ 122p53 mice also develop tumors much faster, and their sarcomas metastasize more rapidly than in p53- animals [21].

Here we report α-enolase, a key glycolytic enzyme in the cytosol that can also be on the cell surface where it is implicated in autoimmune diseases and invasion of transformed cells is regulated as part of the p53 response [23–27].

## Materials and Methods

### Sample collection

The study was conducted with ethical approval from the University of Otago Animal Ethics Committee (approval numbers 20/07 and 21/07). Bone marrow, thymus, and lung tissue were extracted from male mice that were 5–7 weeks of age and homozygous for one of four genotypes: p53+, p53-, mΔ pro [19], or Δ 122p53 [21]. Single cell suspensions were treated with amsacrine (Sigma-Aldrich, St. Louis, MO, USA) or the vehicle control (30% ethanol) for 4.5 hours at 2×10^6^ cells/mL in DMEM supplemented with 20% fetal calf serum, L-glutamine (2 mM), and antibiotics (Life Technologies, Carlsbad, CA, USA). Bone marrow cells were treated with 0.2 μg/mL amsacrine, thymocytes with 1 μg/mL amsacrine, and lung single cell suspensions that were made by enzymatic digestion (collagenase, Sigma-Aldrich, St. Louis, MO, USA) and mechanical dissociation (gentleMACs, Miltenyi Biotec, GmbH, Germany) were treated with 1 μg/mL amsacrine. Preliminary work selected the amsacrine dose and time point post-drug treatment such that p53 was increased before significant apoptosis or cell cycle arrest occurred [19].

For proteasome inhibition, bone marrow cells were pre-treated with the ubiquitin-associated proteasome inhibitor MG132 (5 μM; Sigma-Aldrich, St. Louis, MO, USA) or vehicle control (DMSO) for 90 minutes. Cells were then treated with amsacrine (0.2  μg/mL) and harvested 4 and 6 hours after drug treatment.

Peripheral blood mononuclear cells (PBMCs) were isolated from male mice 5–8 weeks of age. Whole blood was collected in EDTA tubes using density-gradient centrifugation with Histopaque-1083 according to the manufacturer’s instruction (Sigma-Aldrich, St. Louis, MO, USA), and the cell membrane and cytosol fractions isolated for western blotting or placed into short-term cell culture in RPMI medium supplemented with 10% FBS, 10 mM Penicillin Streptomycin, and 10 mM L-glutamine (Life Technologies, Carlsbad, CA, USA). Each PBMCs preparation used blood pooled from 3–10 mice.

Primary tumors were dissected from Δ 122p53 and p53- mice and single cell suspensions made at necropsy by enzymatic digestion with collagenase (Sigma-Aldrich, St. Louis, MO, USA) and mechanical dissociation (gentleMACs, Miltenyi Biotec, GmbH, Germany), and single cells stored in cryoprotective media for isolation of cell membrane and cytosol fractions. All tumors from Δ122p53 had metastasized to at least one other organ, while no metastases were evident in tissues from p53- mice.

### Protein extraction and 2D gel electrophoresis

Cell pellets were solubilized in lysis buffer (30 mM Tris, 7 M urea, 2 Mthiourea, and 4% (w/v) CHAPS, pH 8.0), subjected to sonication, and purified using the 2-D Clean-Up Kit (GE Healthcare, Uppsala, Sweden). Protein concentrations were determined using a Bradford microplate assay (Bio-Rad, Hercules, CA, USA). For each tissue type, an internal standard was prepared using a pooled proteome consisting of an equal amount of protein from each sample for normalization using 2D-DIGE. The pooled internal standard was labeled with Cy3 and the test sample (20 μg) was labeled with Cy5 according to the manufacturer’s instructions (GE Healthcare, Uppsala, Sweden). Samples and standards were separated on 11 cm immobilized pH gradient (IPG) isoelectric focusing (IEF) strips (pH 3–11, non-linear, GE Healthcare, Uppsala, Sweden), followed by second-dimension electrophoresis using Criterion XT 4–12% Bis-Tris precast gels (Bio-Rad, Hercules, CA) [28].

### Image analysis

Following electrophoresis gels were scanned in the Cy3 or Cy5 channel of a Fujifilm FLA-5100 scanner (Fujifilm, Tokyo, Japan) and images analyzed using Delta2D v 4.0 gel analysis software (DECODON, GmbH, Germany). The internal standard image was used to normalize protein spot images between gels. Protein spots were then detected for quantitative and statistical analysis by Delta2D software and the standardized protein spot percentage volume was used to perform statistical tests including univariate (*t*-tests) and multivariate analyses (ANOVA). Protein spots with statistically significant (*P* < 0.05) differential abundance (>1.5-fold) between groups were chosen for identification. Protein spots of interest were located and excised from Coomassie blue-stained preparative gels containing 400 μg of protein lysate as described elsewhere [29]. The excised protein spots were enzymatically cleaved into peptides using trypsin and identified using MALDI-TOF mass spectrometry and peptide mass fingerprinting as previously described [29]. Differentially expressed proteins were further explored by Ingenuity Pathway Analysis (IPA; Ingenuity Systems, Redwood City, CA, USA) to reveal differentially regulated signaling networks and biological processes.

### Membrane and cytosol fractionation

For analysis of cell surface α-enolase expression, cytosol and cell membrane fractions from PBMCs (4x10^6^ cells, three preparations per genotype) and sarcoma tumors (3x10^6^ cells, three tumors per genotype) were obtained using the Calbiochem ProteoExtract Native Membrane Protein Extraction Kit (Merck Millipore, Darmstadt, Germany) or ProteoExtract Transmembrane Protein Extraction Kit (Merck Millipore, Darmstadt, Germany) according to the manufacturer’s instructions.

### Western blotting

Equal amounts of each protein lysate were separated on 4–12% NuPAGE Novex Bis-Tris Mini Gels (Life Technologies, Carlsbad, CA, USA). Unless otherwise stated blots were incubated with antibodies from Abcam (Cambridge, UK) as follows: α-enolase (non neuronal enolase, 1 in 7000, ab49343), beta-actin (1in 7 000, ab82618), ATP5B (ATP synthase subunit beta, mitochondrial, 1 in 700, ab128743), CAPZB (F-actin capping protein subunit beta, 1 in 2 000, ab175212), CD18 (1 in 1 000, ab119830), CD45 (1 in 1 000, ab154885), EEF2 (eukaryotic elongation factor 2, 1 in 1 000, ab33523), EIF4H (eukaryotic translation initiation factor 4H, 1 in 500, ab64323), FAK1 (focal adhesion kinase 1, 1 in 1 000, ab40794), H2B (histone H2B, 1 in 4000, ab52484), HNRNPK (heterogeneous nuclear ribonucleoprotein K, 1 in 1000, ab156570), lamin B1 (1 in 500, ab16048), PSMA1 (proteasome subunit alpha type-1, 1 in 1000, ab3325), PSMC5 (proteasome (prosome macropain) 26S Subunit ATPase 5, 1 in 15 000, ab178681), TKT (transketolase, 1 in 1 000, sc-67120, Santa Cruz Biotechnology, Santa Cruz, CA, USA), TPI1 (trioephosphate isomerase 1, 1 in 6 000, ab28760), TPT1 (transcriptionally controlled tumor protein, 1 in 20 000, ab133568), TAGLN (transgelin, anti-SM22, 1 in 2 000, ab155272), and vimentin (1 in 1 000, ab137321). Alkaline phosphatase secondary antibodies were detected using the WesternBreeze Chemiluminescent Kit detection system according to the manufacturer’s instructions (Life Technologies, Carlsbad, CA, USA), and images captured on X-ray film.

### Immunohistochemistry

Paraffin-embedded cell clot sections were mounted on microscope slides and were subjected to heat-mediated antigen retrieval. Primary antibodies raised against α-enolase (non-neuronal enolase, 1 in 7 000 dilution, ab49343, Abcam, Cambridge, UK) or active caspase-3 (Human/Mouse Caspase-3 Affinity Purified PAb, 1 in 1 000 dilution, R&D Systems, Minneapolis, MN, USA) were detected using the EDL and DAB methods (Dako, Glostrup, Denmark). Alpha-enolase or active caspase-3-positive cells were detected with light microscopy (DM 2000 microscope, DFC 295 camera and Application Suite software, version 3.5.0, Leica, Solms, Germany), and the percentage of positive cells per 500 total cells calculated. The slides were assessed by two authors (AS and NH) who were blinded to the genotype and treatment statuses.

### Gene expression analyses

Total RNA (500 ng) was extracted using Trizol DNase-treated, and cDNA synthesized using oligo-dT primers and SuperScriptIII (Life Technologies, Carlsbad, CA, USA). Quantitative real-time PCR reactions were performed in triplicate using an ABI 7300 Real-Time PCR System and SYBR Green PCR Master Mix (Life Technologies, Carlsbad, CA, USA). The comparative C_T_ method was used for the comparative quantification of gene expression, with results from each time point normalized against *beta-2 microglobulin* (*beta2-M)* expression. Primer sequences for *ENO1* and *beta2-M* were those used previously [19, 30].

### ELISA

Tumor necrosis factor alpha (TNF-α) concentrations in media were quantified in short-term cultured PBMCs (2x10^6^ cells/mL) using the Quantikine ELISA Mouse TNF-α immunoassay according to the manufacturer’s instructions (R&D Systems, Minneapolis, NE, USA). For plasminogen stimulation, plasminogen (Lys-plasminogen, 20 μg/mL) was added to PBMCs cell culture incubations and tissue plasminogen activator (3 nM) added prior to TNF-α measurement (Sigma-Aldrich, St. Louis MO, USA). For inhibition of nuclear factor kappa-light-chain-enhancer of activated B cells (NF-kappaB), PBMCs were pre-treated for 90 minutes with the inhibitor BAY11–7082 (2.5 μM, Sigma-Aldrich, St. Louis MO, USA) or DMSO (vehicle control) prior to stimulation with plasminogen. To inhibit plasminogen activation to plasmin tranexamic acid (TXA, 10 mM, Sigma Aldrich, St. Louis, MO, USA) was added. To increase the amount of alpha-enolase on the cell surface 5 μg/mL of bacterial lipopolysaccharide (LPS, Sigma-Aldrich, St. Louis MO, USA) was added for 6 hours [24]. To block plasminogen binding to alpha-enolase polyclonal antibodies raised against alpha-enolase (non-neuronal enolase, ab49343, Abcam, Cambridge, UK and enolase 1 antibody ThermoFisher Scientific, Waltham, MA, US, 15 μg/mL each) or rabbit IgG (Sigma-Aldrich, St. Louis MO, USA and as a control) were added to PBMCs following LPS incubation (5 μg/mL for 6 hours).

### Statistical analysis

In addition to those already stated, TNF-alpha concentration differences were compared between genotypes and/or treatment. The data are expressed as the mean ± SD, and the statistical significance was determined between the experimental groups using one-way analysis of variance (ANOVA) followed by Tukey’s multiple comparisons test. Paired data were analyzed using the paired two-tailed Student’s t test. *P* < 0.05 was considered statistically significant and GraphPad Prism software, version 6.00 for Macintosh (GraphPad Software, San Diego, CA, USA) was used.

## Results

### A wild-type p53 response showed the greatest global change in the proteome pattern with tissue-specific differences

The proteomic analysis of bone marrow and thymus using 2D-DIGE showed the greatest number of statistically significant spots (ANOVA, *P* < 0.05) in drug-treated p53+ cells compared to all other treated and untreated cells. In bone marrow p53+ treated cells a large number of proteins had statistically significant (ANOVA, *P* < 0.5) reduced abundance when compared with treated cells from the other genotypes (S1A Fig.), and in the thymus many proteins had statistically significant (ANOVA, *P* < 0.5) increased protein abundance in treated p53+ cells compared with treated cells from the other genotypes (S2A Fig.).

Unlike p53+ cells, unsupervised hierarchical clustering of treated and untreated mutant or p53- did not show distinct clusters suggesting that the p53-, mΔpro, and Δ122p53 treated cells had no global changes in the proteome pattern upon amsacrine treatment and instead had similar patterns to p53+ untreated cells (data not shown). The mΔpro and Δ122p53 mutants were most similar and clustered in induced and un-induced conditions.

In lung cells hierarchical clustering of statistically significant spots did not show a distinct cluster amongst the different models (data not shown). This result is consistent with other studies that show organs with a lower proliferation rate are less sensitive to p53-dependent genotoxic stress [31,32].

### Targets of ubiquitin C signaling are altered with a wild-type p53 response

Mass spectrometry of 2D-DIGE spots of interest identified 31 proteins (Table 1). Twenty-six proteins were associated with a wild-type p53 response, including 10 proteins that were decreased in bone marrow, 13 that were increased in thymus, and 4 that were differentially expressed in lung.

**Table 1:** List of proteins differentially regulated amongst different p53 genotypes.

Protein	Findings from the current study	Accession number	Overall Functions	Established relationships with p53
Bone marrow				
Proteasome (Prosome Macropain) 26S Subunit, ATPase, 5 (PSMC5):	Decreased in p53+ treated	P62196	Protein turnover, negative regulation of transcription.	26S proteasome involved in degradation of ubiquitinated p53 [54]
40S ribosomal protein SA (RPSA):	Decreased in p53+ treated	P14206	40S ribosomal subunit assembly, laminin receptor, cell fate determination and tissue morphogenesis.	
Actin-related protein 2/3 Complex, Subunit 5, 16 kDa (ARPC5):	Decreased in p53+ treated	Q9CPW4	Actin polymerization, cilium biogenesis/degradation	
Alpha enolase:	Decreased in p53+ treated Increased in Δ 122p53	P17182	Multifunctional enzyme, glycolysis, growth control, hypoxia tolerance, inflammation*	
Creatine kinase M-type (CKM):	Decreased in p53+ treated	P07310	Phosphocreatine biosynthesis	
Eukaryotic translation initiation factor 4H (EIF4H):	Decreased in p53+ treated	Q9WUK2	Protein biosynthesis	
F-actin capping protein subunit beta (CAPZB):	Decreased in p53+ treated	P47757	Actin filament growth, cell morphology and cytoskeletal organization	Interacts with p53 [55]
Ferritin light chain 1:	Decreased in mΔ pro treated	P29391	Iron storage and homeostasis	
Plastin-2 (LCP1):	Decreased in p53+ treated	Q61233	T-cell activation, actin filament bundle assembly, organ regeneration, and intracellular protein transport	
Proteasome subunit alpha type-1 (PSMA1):	Decreased in p53+ treated	Q9R1P4	Protein turnover, negative regulation of inflammation	26S proteasome involved in degradation of ubiquitinated p53 [54]
T-complex protein 1 subunit epsilon (CCT5) :	Decreased in Δ 122p53	P80316	Molecular chaperone, and ciliogenesis	p53 binds to CCT complex, required for correct p53 folding [56]
Transgelin (TAGLN):	Increased in Δ 122p53	P37804	Organ development	
Triosephosphate isomerase (TPI1)	Decreased in p53+ treated	P17751	Gluconeogenesis, glycolysis, pentose phosphate pathway	
Thymus				
Annexin A5 (ANXA5):	Increased in p53+ treated Decreased in Δ 122p53	P48036	Anticoagulant	
ATP synthase subunit beta, mitochondrial (ATP5B):	Increased in p53+ treated	P56480	ATP synthesis, ion transport	
Elongation factor 2 (EEF2):	Increased in p53+ treated	P58252	Protein biosynthesis	Interacts with p53 [57]
F-actin capping protein subunit beta (CAPZB):	Increased in p53+ treated	P47757	Actin filament growth, cell morphology and cytoskeletal organization	Interacts with p53 [55]
Heat shock protein 90kDa alpha (cytosolic), class B member 1 (HSP90-beta)	Increased in Δ 122p53, mΔ pro, and p53-	P11499	Molecular chaperone	Interacts with p53 [58]. p53 reduces *HSP90AB1* expression [59]
Heterogeneous nuclear ribonucleoprotein K (HNRNPK):	Increased in p53+ treated Increased in Δ 122p53 untreated	P61979	Transcription regulation, and mRNA processing and splicing	Transcriptional co-factor for p53 mediated gene transcription [60, 61, 63]
Lamin-B1 (LMNB1):	Increased in p53+ treated	P14733	Regulation of JNK signaling and G2/M procession	Senescence induced by Lamin B is reversed upon p53 inactivation [62]
Proteasome subunit alpha type-1 (PSMA1):	Increased in p53+ treated	Q9R1P4	Protein turnover, negative regulation of inflammation	26S proteasome involved in degradation of ubiquitinated p53 [54]
Proteasome subunit alpha type-3 (PSMA3):	Increased in p53+ treated	O70435	Protein turnover	26S proteasome involved in degradation of ubiquitinated p53 [54]
Proteasome subunit beta type 8 (PSMB8):	Increased in p53+ treated	P28063	Antigen processing and presentation, and proteolysis	26S proteasome involved in degradation of ubiquitinated p53 [54]
Transketolase (TKT):	Increased in Δ 122p53	P40142	Pentose phosphate pathway	
Translationally-controlled tumor protein (TPT1):	Decreased in p53+ treated. Increased in Δ 122p53	P63028	Allergy response, anti-apoptosis, and cell cycle regulation	Interacts with p53 [63, 64]. Is a transactivation target of p53 [64]
Tropomyosin alpha-1 chain (TPM1):	Increased in p53+ treated	P58771	Cardiac muscle contraction, and stabilizing cytoskeleton actin filaments	
14–3–3 protein epsilon(14–3–3E):	Increased in Δ 122p53, mΔ pro, and p53-	P62259	Adapter protein	14–3–3 proteins interact with p53 [65, 66]
14–3–3 protein eta	Increased in p53+ treated	P68510	Adapter protein	
14–3–3 protein gamma	Increased in p53+ treated	P61982	Adapter protein	
14–3–3 protein zeta/delta	Increased in p53+ treated	P63101	Adapter protein	
Lung				
Aldehyde dehydrogenase mitochondrial (ALDH2):	Decreased in p53+ treated Increased in Δ 122p53	P47738	Metabolism	
26S proteasome non-ATPase regulatory subunit 3 (PSMD3):	Increased in p53+ treated	P14685	Protein turnover	26S proteasome involved in degradation of ubiquitinated p53 [54]
Destrin	Increased in p53+ treated	Q9R0P5	Actin filament depolymerization	
Valosin containing protein (VCP):	Decreased in p53+ treated. Increased in Δ 122p53	Q01853	DNA damage and repair	Interacts with p53 [48]

Proteins were increased or decreased at least 1.5 fold in comparison to p53 wild-type (p53+) untreated cells from the bone marrow, thymus, or lung tissue. Proteins were identified from 2D gel electrophoresis separation and MALDI-TOF mass spectrometry and peptide mass fingerprinting. *, cell surface expression; in bold, proteins selected for validation using western blotting.

**Figure 1:**
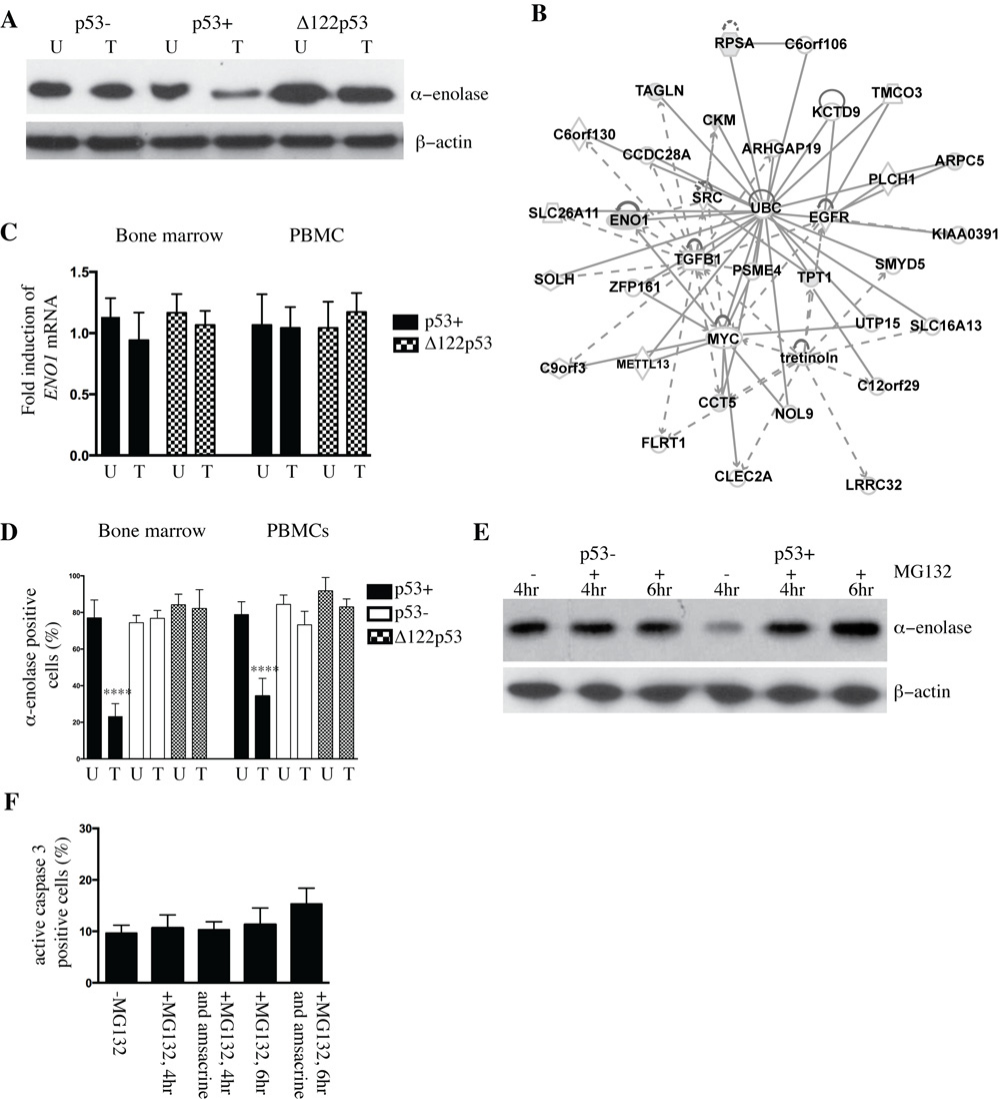
Altered abundance of alpha-enolase in p53+ and Δ122p53 bone marrow. **A.** Alpha-enolase is decreased in wild-type p53 (p53+) bone marrow upon DNA damage with amsacrine treatment and increased in Δ122p53 bone marrow cells irrespective of amsacrine treatment compared with p53 null (p53-) cells by western blotting. Amsacrine treated (T) or untreated (U). **B.** Network analysis of the differentially expressed proteins in p53+ bone marrow untreated and p53+ bone marrow treated with amsacrine from two-dimensional fluorescence difference gel electrophoresis using Ingenuity Pathways Analysis software. Gray shading indicates proteins identified in the current study. Solid and dashed lines indicate direct and indirect interactions, respectively. **C.** The decrease in α-enolase in p53+ treated bone marrow was not due to reduced expression of *ENO1*. No significant differences were found in the amounts of *ENO1* transcript expressed in p53+ and Δ122p53 bone marrow and peripheral blood mononuclear cells (PBMCs) in untreated cells (U) and cells treated with amsacrine (T) using real-time PCR. The results are expressed as the mean ± SD, from 3 mice per genotype and represent the fold increase in *ENO1* expression, normalized for *beta2-M* expression. **D.** The percentage of alpha-enolase positive cells was reduced in p53+ bone marrow and peripheral blood mononuclear cells (PBMCs) treated with amsacrine. Bone marrow and PBMCs from five mice per genotype were extracted and treated with amsacrine (T) or left untreated (U). Following short-term culture, cells were fixed and cell clots sectioned. Alpha-enolase was detected using immunohistochemistry. Positive cells were identified by light microscopy and the percentage of positive cells per total cell count (500 cells) was compared between treated and untreated cells; the results are expressed as the mean ± SD (n = 6 mice per genotype), ****, *P* < 0.0001. **E.** The ubiquitin-associated proteasome inhibitor MG132 was added to p53+ and p53- bone marrow treated with amsacrine for 4 or 6 hours. Post-treatment, the amount of alpha-enolase in cell lysates was compared between cells treated with amsacrine or left untreated, by western blotting. **F.** MG132 treatment did not lead to a statistically significant increase in the percentage of apoptotic cells in p53+ bone marrow at 4 and 6 hours post-treatment with MG132 alone or with MG132 and amsacrine. Apoptotic cells were determined from counting the percentage of active caspase-3-positive cells in bone marrow from immunohistochemistry-stained sections and light microscopy. The percentage of positive cells per total cell count (500 cells) was compared between treated and untreated cells; the results are expressed as the mean ± SD (n = 3).

Reduced expression in p53+ treated bone marrow was validated for 8 proteins by western blot analysis (Table 1). These proteins included those involved in metabolism alpha-enolase (Fig. 1A), TPI1 (S1B Fig.), and CKM (creatine kinase M-type, data not shown) proteins involved in protein synthesis and turnover PSMA1, PSMC5, and EIF4H (S1B Fig.), and those involved in actin rearrangement CAPZB (S1B Fig.) and plastin-2 (data not shown).

Proteins reduced in p53+ treated bone marrow fitted into two networks using Ingenuity software (Ingenuity Systems, http://www.Ingenuity.com). Many proteins are targets of ubiquitin C (network score 37, Fig. 1B), and 5 proteins composed a second network with myc and transforming growth factor beta as key nodes. The most significant canonical pathway that was significantly over represented was glycolysis I, followed by creatine-phosphate biosynthesis, sucrose degradation V, and gluconeogenesis I (*P* < 0.05). The disease and disorder analysis showed the strongest correlation with hematological disease, followed by immunological disease and inflammatory disease (*P* < 0.05).

For validation of proteins in the thymus, western blotting showed increased protein abundance for 8 proteins in p53+ treated cells (Table 1 and examples given in S2B and S2C Fig.). These proteins included those involved in metabolism, such as ATP5B, protein biosynthesis and protein turnover EEF2, PSMA1, PSMA3, and PSMB8, and those involved in cell cycle progression such as lamin B1.

Proteins differentially expressed in thymus cells fitted into three networks using Ingenuity software. All proteins are targets of ubiquitin C (score 31). Proteins altered in p53+ treated cells compared with untreated cells formed a network of four proteins centred on myc and tumor necrosis factor (TNF) nodes (S2D Fig.). A comparison of proteins altered in p53+ treated cells compared with p53-, mΔpro, and Δ122p53 treated cells were centred on a network of YWHAZ (tyrosine 3-monooxygenase/tryptophan 5-monooxygenase activation protein, zeta polypeptide) signaling (score 41). The most significant canonical pathway in this network was p70s6k signaling, followed by P13K/AKT signaling, and cell cycle: G2/M DNA damage checkpoint regulation (*P* < 0.05). The disease and disorder analysis showed the strongest correlation with cancer (*P* < 0.05).

Reduced alpha-enolase as part of a p53+ response was further investigated. The decrease in alpha-enolase in p53+ treated bone marrow and PBMCs was not due to reduced expression of *ENO1* as determined by real-time PCR (Fig. 1C). There was no difference in *ENO1* expression between p53+, and Δ122p53 untreated and treated cells (Fig. 1C). Reduced alpha-enolase in p53+ treated bone marrow was also evident from immunohistochemistry of bone marrow and PBMCs following amsacrine treatment (Fig. 1D). The percentage of alpha-enolase positive cells was decreased in p53+ treated bone marrow (*P* < 0.0001) and PBMCs (*P* < 0.0001) compared with p53+ untreated cells and treated and untreated p53- and Δ122p53 cells. Representative photomicrographs to illustrate a reduction in alpha-enolase positive cells in p53+ treated bone marrow compared to p53+ untreated bone marrow are shown in S3 Fig.

To investigate the importance of ubiquitin-associated proteasome degradation for reduced abundance of alpha-enolase, bone marrow from p53+ and p53- mice was pre-treated with the ubiquitin-associated proteasome inhibitor MG132 prior to amsacrine treatment and the abundance of alpha-enolase determined by western blotting. The addition of MG132 to p53+ cells effectively stabilized alpha-enolase at 4 and 6 hours post-amsacrine treatment compared to p53+ cells that were treated with amsacrine alone (Fig. 1E). No effect on alpha-enolase was evident in p53- cells in the presence of MG132 (Fig. 1E). Treatment with MG132 can increase apoptosis [33]. To test whether an increase in apoptosis with MG132 treatment was a confounding factor, the percentage of active caspase-3-positive cells was counted in p53+ bone marrow following treatment with MG132 alone or the combined treatment of MG132 and amsacrine. No significant differences were found in apoptotic cells after 4 or 6 hours with MG132 alone or when combined with amsacrine in comparison with untreated cells cultured for 4 hours. There was a trend toward increased apoptotic cells at 6 hours with MG132 and amsacrine treatment; however, the difference was not statistically significant (Fig. 1F).

In summary, the reduction in proteins upon a p53+ response to DNA damage is at least in part due to ubiquitin-associated proteasome degradation.

### Proteins overexpressed in cancer are altered in 122p53 cells

Although 122p53 bone marrow, thymus, and lung showed no global changes in protein abundance compared with the other mutants and p53+ untreated cells, individual proteins showed aberrant abundance (Table 1). Seven of these proteins were validated by western blotting to identify possible mechanisms by which 122p53 promotes tumorigenesis. In bone marrow α-enolase was increased. In thymocytes (S2C Fig.) TKT and TPT1 were increased in untreated and treated 122p53 cells. Heterogeneous nuclear ribonucleoprotein K was increased in 122p53 untreated cells (S2C Fig.). In the lung valosin-containing protein (VCP) was increased in 122p53 cells (data not shown).

### Alpha-enolase is increased on the Δ122p53 mononuclear membrane

Other studies have established a link between overexpression of α-enolase and increased α-enolase on the cell surface [24]. This link, and the finding of increased α-enolase in Δ122p53 cells, was the basis for determining whether α-enolase was increased on the surface of Δ122p53 PBMCs.

**Figure 2:**
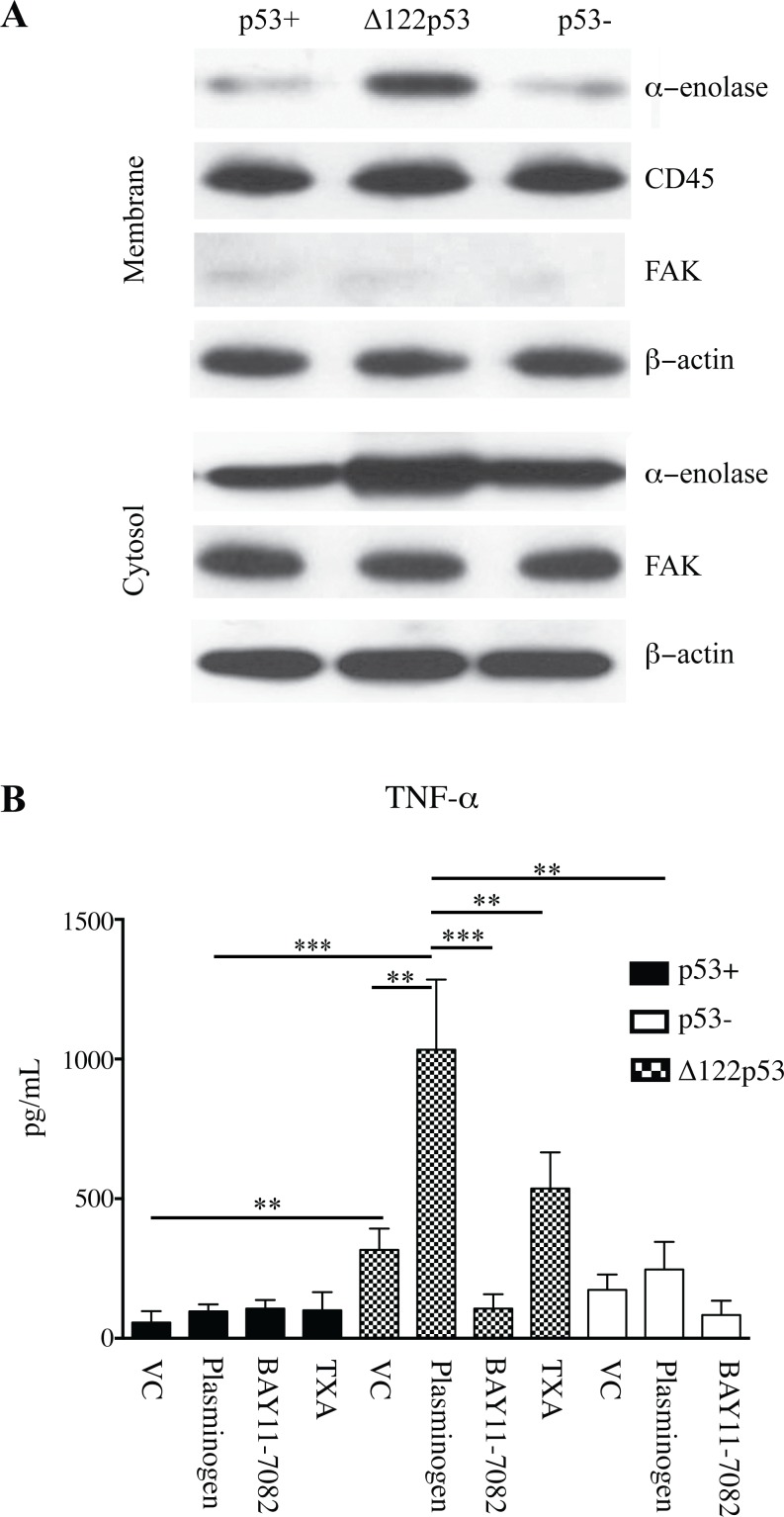
Increased alpha-enolase on the Δ122p53 PBMC cell membrane. **A**. Increased alpha-enolase is present on the cell membrane of Δ122p53 PBMCs compared to that on p53- and p53+ PBMCs. The cell membrane and cytosolic fractions of untreated PBMCs from p53+, p53-, and Δ122p53 mice were separated and subjected to western blotting with an antibody to alpha-enolase. β-actin, FAK, and CD45 were used as loading controls for total protein, cytosolic, and cell membrane fractions, respectively. **B**. Increased TNF-alpha was released from Δ122p53 PBMCs following plasminogen stimulation compared with that from p53+ and p53- PBMCs. PBMCs were pre-incubated with plasminogen (lys-plasminogen) with or without the NFκB inhibitor BAY 11–7082 (2.5 μM for 90 minutes), or the inhibitor to plasmin activation (TXA, 10 mM), or vehicle-treated only (VC). Following pre-incubation and prior to TNF-alpha measurement, tissue plasminogen activator (3 nM) was added, and the amount of TNF-alpha secreted in culture media was measured by ELISA. The results represent the mean ± SD (n = 3 for each measurement). ***, *P* < 0.001, **, *P* < 0.01, *P* < 0.05.

PBMCs were separated into cytosol and cell membrane fractions and the amount of alpha-enolase in each fraction measured using western blotting. Increased alpha-enolase was present in the membrane and cytosolic fractions of Δ122p53 cells (Fig. 2A) compared with p53+ and p53- cells in 3 separate experiments. p53+ and p53- cells showed minimal alpha-enolase in the cell membrane (Fig. 2A). These results suggested that the Δ122p53 allele led to increased alpha-enolase in the cytosol and cell membrane.

### Δ122p53 mononuclear cells contribute to a pro-inflammatory response with plasminogen activation

Increased alpha-enolase on the cell surface of Δ122p53 PBMCs would be expected to elicit a pro-inflammatory response to plasminogen stimulation [24–26]. To determine if this occurs, Δ122p53, p53+, and p53- PBMCs were pre-treated with plasminogen, and the concentration of the pro-inflammatory cytokine TNF-alpha released into culture media measured by ELISA (Fig. 2B). Δ122p53 PBMCs had increased TNF-alpha concentrations compared with p53+ and p53- cells with and without plasminogen induction. In the vehicle control treated cells the concentration of TNF-alpha was 5.6-fold higher in Δ122p53 PBMCs compared to that in p53+ (*P* < 0.01) and 2-fold higher compared to p53- cells (*P* < 0.05). Following plasminogen treatment the concentration of TNF-alpha increased 3.3-fold in Δ122p53 PBMCs compared to that treated with the vehicle control alone (from 317 ± 76 pg/mL without plasminogen to 1033 ± 252 pg/mL with plasminogen treatment, *P* < 0.01). The induction of TNF-alpha following plasminogen treatment in Δ122p53 PBMCs required plasminogen to plasmin activation, as evidenced by a reduction in plasminogen-induced TNF-alpha expression upon co-treatment with the plasmin activation inhibitor TXA (Fig. 2B, *P* < 0.01).

In p53+ cells, plasminogen treatment did not induce TNF-alpha compared with cells treated with the vehicle control. In p53- cells a trend toward increased TNF-alpha occurred that did not reach a statistically significant level.

### The pro-inflammatory response of Δ122p53 involved NF-kappaB signaling

The induction of TNF-alpha by plasminogen can involve NF-kappaB signaling [25]. To test whether the increased TNF-α in Δ122p53 PBMCs was due to NF-κB signalling, Δ122p53 PBMCs were pre-treated with the NF-κB inhibitor BAY11–7082. Results showed that the increase in TNF-alpha concentration following plasminogen treatment was obliterated upon NF-κB inhibition, *P* < 0.001 (Fig. 2B). Overall, these data suggest that increased TNF-alpha expression in Δ122p53 cells involved NF-κB signaling.

When a similar experiment was performed with p53+ cells incubation with BAY11–7082 did not reduce TNF-alpha concentrations, which remained similar to those in cells treated either with the vehicle control or plasminogen (Fig. 2B). In p53- cells BAY11–7082 treatment did not alter TNF-alpha concentrations compared with those treated with the vehicle control only. However, BAY11–7082 treatment did reduce TNF-alpha concentrations in cells treated with plasminogen (*P* < 0.01). This suggests that NF-κB induction of TNF-alpha suppressed most robustly by a wild-type p53 response.

**Figure 3:**
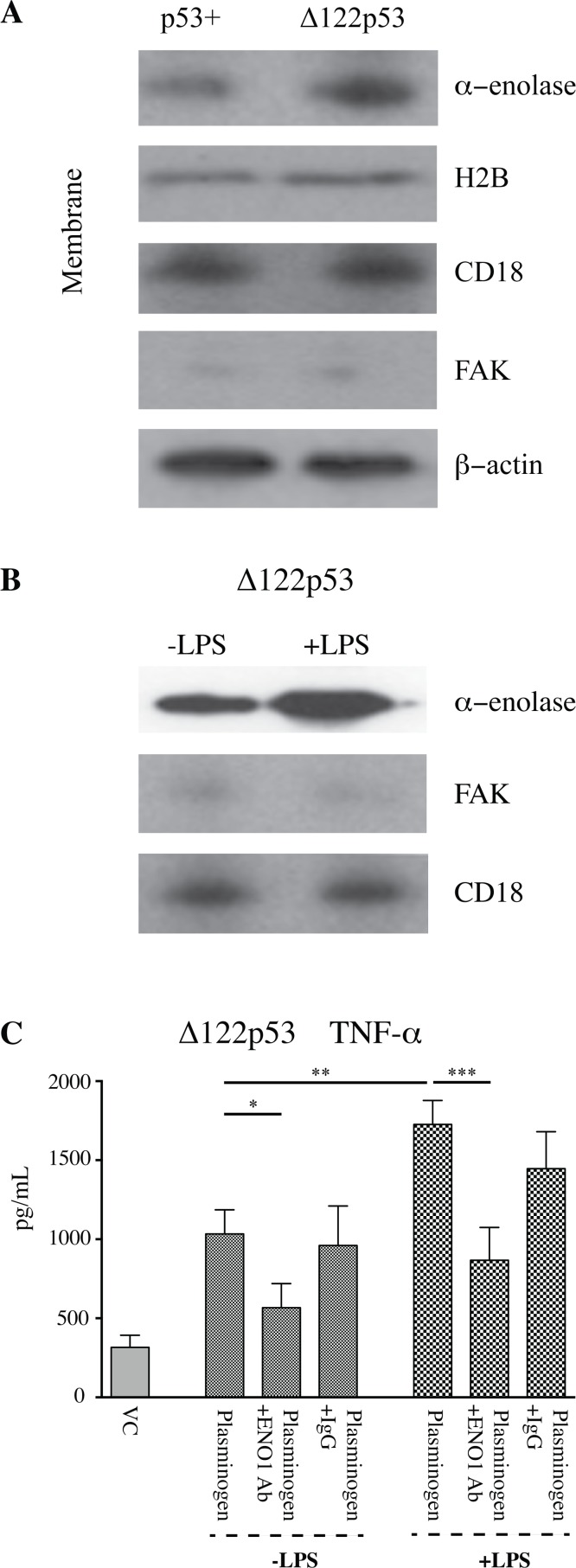
Increased α-enolase function on the Δ122p53 cell membrane. **A.** Alpha-enolase was not the only plasminogen receptor increased on the Δ122p53 cell membrane. The cell membrane and cytosolic fractions of untreated PBMCs from p53+ and Δ122p53 mice were separated and subjected to western blotting with an antibody to histone H2B and alpha-enolase. β-actin, FAK, and CD18 were used as loading controls for total protein, cytosolic, and cell membrane fractions, respectively. **B.** LPS was added to enhance the amount of alpha-enolase on the Δ122p53 PBMCs cell membrane. The cell membrane and cytosolic fractions of untreated Δ122p53 PBMCs or those incubated with LPS (5 μg/mL for 6 hours) were separated and subjected to western blotting with an antibody to alpha-enolase. FAK and CD18 were used as loading controls for cytosolic and cell membrane fractions, respectively. **C.** TNF-alpha released from Δ122p53 following pre-incubation with lys-plasminogen and LPS (5 μg/mL for 6 hours) with or without two anti-alpha-enolase antibodies (each at 15 μg/mL) to block plasminogen binding or rabbit IgG as a control (30 μg/mL). Prior to TNF-alpha measurement, tissue plasminogen activator (3 nM) was added and the amount of TNF-alpha secreted in culture media was measured by ELISA. The results represent the mean ± SD (n = 3 for each measurement) ***, *P* < 0.001, **, *P* < 0.01.

Alpha-enolase may not be the only plasminogen receptor increased at the Δ122p53 cell surface. Western blots for an alternative plasminogen receptor, histone H2B, using the separated transmembrane cellular component showed a slight increase in histone H2B at the Δ122p53 compared to that on the p53+ PBMC cell membrane (Fig. 3A) [34]. To determine that the increased TNF- alpha in Δ122p53 PBMCs following plasminogen stimulation was due to alpha-enolase, the TNF-alpha measurements were repeated using Δ122p53 PBMCs incubated with LPS in addition to plasminogen to increase the amount of alpha-enolase on the Δ122p53 PBMC cell surface and in separate incubations antibodies toward alpha-enolase where included to block plasminogen binding. The addition of LPS did lead to increased alpha-enolase on the Δ122p53 PBMCs cell surface (Fig. 3B), and to increased TNF-alpha compared with Δ122p53 PBMCs incubated without LPS (Fig. 3C, *P* < 0.01). A reduction in TNF-alpha was also found in the presence of alpha-enolase antibodies in Δ122p53 PBMCs, with no reduction in TNF-alpha found with the IgG control (Fig. 3C, *P* < 0.001).

These results are consistent with increased alpha-enolase function as a plasminogen receptor on the Δ122p53 cell membrane.

### Δ122p53 tumors also show increased alpha-enolase on the cell membrane

**Figure 4:**
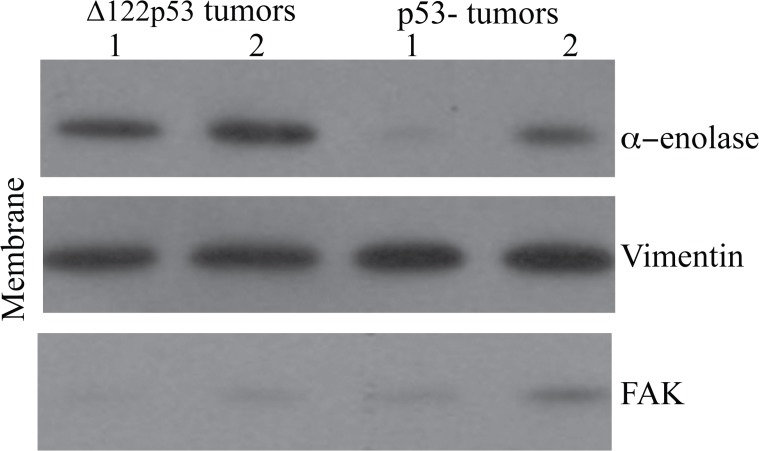
Increased α-enolase on the Δ122p53 tumor cell membrane. Tumors from Δ122p53 mice had increased alpha-enolase at the cell surface compared to tumors from p53- mice. Sarcomas were dissected from Δ122p53 and p53- mice at necropsy, the cytosolic and cell membrane fractions were separated, and these fractions were subjected to western blotting using an antibody to alpha-enolase. The cell membrane results from two tumors per genotype (1 and 2) are shown. Western blotting for vimentin was used as a loading control for the mesenchymal cell membrane fraction and FAK was included to control for cytosolic contamination in cell membrane preparations.

Increased plasmin activity followed by extracellular matrix degradation as a result of increased alpha-enolase on the Δ122p53 tumor cell surface would provide an explanation for why Δ122p53 sarcomas metastasize more rapidly compared to those from p53- mice. The cytosolic and membrane fractions were separated in 6 sarcomas, 3 from Δ122p53 and 3 from p53- mice. The amount of alpha-enolase in each fraction was measured using western blotting. Increased alpha-enolase was present in the membrane fraction of all 3 tumors from Δ122p53 mice compared with those from p53- mice. The results from 2 tumors per genotype are shown in Fig. 4. The finding of increased alpha-enolase on the Δ122p53 tumor cell surface is supportive of alpha-enolase having a role in tumor invasion in the Δ122p53 model.

## Discussion

The results of the complete proteome in response to DNA damage obtained from this study highlight p53-directed protein changes rather than transcript responses. This has enabled us to discover more about how p53 responds to stress and show for the first time that p53 function affects the amount of alpha-enolase in the cell. A wild-type p53 stress response led to a reduction in alpha-enolase. A mimic for the human Δ133p53α isoform showed that this isoform might increase alpha-enolase, leading to plasminogen activation and increased pro-inflammatory cytokine production.

Alpha-enolase is found in almost all tissues and is one of the most abundantly expressed proteins in the cytosol, where it is best known for its role in glycolysis converting 2-phosphoglycerate to phosphoenolpyruvate [35]. It is commonly associated with differential expression in proteomic studies, which reveal that alpha-enolase is often altered when normal and diseased tissues are compared [36]. The results of this study suggest alpha-enolase is often found in proteomic studies due to different p53 states being compared. Restricting alpha-enolase activity is consistent with the well-demonstrated role of wild-type p53 as a tumor suppressor. Alpha-enolase provides a metabolic advantage to compensate for hypoxia and is overexpressed in many cancer types [27,37–40].

The reduction in alpha-enolase following a wild-type p53 DNA damage response was eliminated on inhibition of ubiquitin C, suggesting p53 reduces alpha-enolase by targeting it toward ubiquitin C-mediated degradation. In the current study the protein reported as alpha-enolase using western blotting corresponded to a size of approximately 48 kDa, which is consistent with the size of alpha-enolase and not that of the smaller-sized protein, myc-binding protein-1. That protein is also encoded by the *enolase 1 alpha (ENO1)* gene and has been shown by others to be degraded by ubiquitin-dependent degradation [41,42]. The amsacrine dose and the time point following treatment were chosen specifically to identify changes that occur before p53-directed apoptosis or cell cycle arrest was evident. Consistent with this, the immunohistochemistry analysis showed decreased alpha-enolase staining in intact cells in DNA-damaged p53+ bone marrow and PBMCs. Therefore, the reduction in alpha-enolase observed in p53+ treated cells was unlikely to be due to increased apoptosis or necrosis of p53+ treated cells. The reduction in alpha-enolase in p53+ treated cells was not due to reduced *ENO1* transcript expression. It is possible that the reduction in alpha-enolase results from processes other than ubiquitin C-mediated degradation such as changes to protein synthesis.

The increased alpha-enolase in the Δ133p53alpha mimic suggests p53 isoforms may have a different function from wild-type p53 in regulating alpha-enolase and may function to increase alpha-enolase abundance. We anticipate the increased response to plasminogen stimulation on the Δ122p53 sarcoma cell surface would lead to increased extracellular matrix degradation and invasion of transformed cells. This provides a possible mechanism to explain why Δ122p53 sarcomas metastasize, whereas sarcomas from p53- mice rarely do [21]. Increased migration of hemopoietic cells by alpha-enolase activity on the Δ122p53 hemopoietic cell surface may also explain another feature of the Δ122p53 mouse model in which aberrant lymphocyte aggregates occur in the lung and liver [21].

Alpha-enolase is colocalized on the cell surface with urokinase plasminogen activator receptor, urokinase, and plasminogen, and the close association of these proteins was suggested to be responsible for presenting alpha-enolase on the cell surface [26]. Currently the mechanism and stimulus for increased alpha-enolase in Δ122p53 cells is unknown. Increased *ENO1* expression in Δ122p53 spleen [21] or bone marrow was not evident by microarray analysis (data not shown) or by real-time PCR in the current study, which demonstrated the increased power provided by proteomic studies. However, Δ122p53 mice did have increased expression of genes involved in immune function including antigen presenting, leukocyte activation, and inflammation mediated by chemokine and cytokine signaling [21]. Increased pro-inflammatory cytokine signaling may increase alpha-enolase on the surface of PBMCs [25]. In the current study Δ122p53 PBMCs showed increased TNF-alpha levels before stimulation with plasminogen, a result consistent with our previous study that reported multiple pro-inflammatory cytokines were increased in Δ122p53 serum [21]. The NF-κB pathway was involved in inducing TNF-α alpha as little TNF-α was released in Δ122p53 PBMCs when NF-κB signaling was inhibited, suggesting Δ122p53 may function to increase NF-κB signaling.

In healthy PBMCs alpha-enolase can be transported to the cell surface upon treatment with LPS, suggesting cytosolic alpha-enolase is recruited to the cell surface as part of a response to pathogens [24]. p53 isoforms have been postulated as a key regulator in the initial response to viral and bacterial pathogens [18,43–45]. In *Heliocobacter pylori*-infected cells Δ133p53α induced NF-κB target genes including pro-inflammatory cytokines by inhibiting IκBα activity [18]. Whether Δ122p53 inhibited IκBα was not established in the current study. A study by Wei *et al*. 2012, demonstrated that the induction of NF-κB target genes by Δ133p53α in response to *H*. *Pylori* infection required the presence of wild-type p53 [18]. This highlights a limitation of the Δ122p53 model. Although the model can offer insight into the functions of the Δ133p53α protein and identify cell types of interest to explore Δ133p53α function, the expression of Δ122p53 it is not under the same regulation as Δ133p53α. In addition, in human cells Δ133p53α functions along with wild-type p53 and other p53 isoforms. The interplay between Δ133p53α and other forms of p53 was not part of the current study.

Alpha-enolase was not the only protein increased in Δ122p53 cells, and it could be another protein that may be responsible for increased NF-κB signaling. Valosin-containing protein and HNRNPK were both increased in Δ122p53 cells and each enhances NF-κB signaling [46,47]. These proteins and TPT1, also increased in Δ122p53 cells, have other tumorigenic properties. In non-small cell lung carcinoma cells VCP activity decreased p53 function [48]. Heterogeneous nuclear ribonucleoprotein K promoted tumor metastasis and angiogenesis and interacted with mutant p53 in pancreatic cancer cells [49,50], and TPT1 has anti-apoptotic properties and its down-regulation has been implicated in cancer reversion [51–53].

## Conclusions

Together, the current study suggests that different p53 functions regulate alpha-enolase. A wild-type p53 response to DNA damage reduces alpha-enolase and adds to the tumor suppressor repertoire of p53. However, p53 isoforms may increase α-enolase and promote plasminogen signaling followed by inflammation and invasion.

The multifactorial functions of α-enolase are growing and reducing α-enolase on the cell surface may be important to limit other tumorigenic functions. This includes a role for α-enolase as a plasminogen receptor linked to increased cellular migration, inflammation, and invasion [23–26].

## Supporting Information

S1 Fig Changes in protein abundance with a wild-type p53 response in bone marrow. (TIF)

S2 Fig Changes in protein abundance with a wild-type p53 response in thymus. (TIF)

S3 Fig Changes in protein abundance with a wild-type p53 response in thymus. (TIF)

## References

[1] Oren M (2003) Decision making by p53: life, death and cancer. Cell Death Differ 10: 431–442. 12719720 10.1038/sj.cdd.4401183

[2] Levine AJ, Tomasini R, McKeon FD, Mak TW, Melino G (2011) The p53 family: guardians of maternal reproduction. Nat Rev Mol Cell Biol 12: 259–265. 10.1038/nrm3086 21427767

[3] Pant V, Quintas-Cardama A, Lozano G (2012) The p53 pathway in hematopoiesis: lessons from mouse models, implications for humans. Blood 120: 5118–5127. 10.1182/blood-2012-05-356014 23018641 PMC3537308

[4] Quadrato G, Di Giovanni S (2012) Gatekeeper between quiescence and differentiation: p53 in axonal outgrowth and neurogenesis. Int Rev Neurobiol 105: 71–89. 10.1016/B978-0-12-398309-1.00005-6 23206596

[5] Maddocks OD, Vousden KH (2011) Metabolic regulation by p53. J Mol Med (Berl) 89: 237–245. 10.1007/s00109-011-0735-5 21340684 PMC3043245

[6] Hoesel B, Schmid JA (2013) The complexity of NF-kappaB signaling in inflammation and cancer. Mol Cancer 12: 86 10.1186/1476-4598-12-86 23915189 PMC3750319

[7] Surget S, Khoury MP, Bourdon JC (2013) Uncovering the role of p53 splice variants in human malignancy: a clinical perspective. Onco Targets Ther 7: 57–68. 10.2147/OTT.S53876 24379683 PMC3872270

[8] Bourdon JC, Fernandes K, Murray-Zmijewski F, Liu G, Diot A, et al (2005) p53 isoforms can regulate p53 transcriptional activity. Genes Dev 19: 2122–2137. 16131611 10.1101/gad.1339905 PMC1221884

[9] Flaman JM, Waridel F, Estreicher A, Vannier A, Limacher JM, et al (1996) The human tumour suppressor gene p53 is alternatively spliced in normal cells. Oncogene 12: 813–818. 8632903

[10] Yin Y, Stephen CW, Luciani MG, Fahraeus R (2002) p53 Stability and activity is regulated by Mdm2-mediated induction of alternative p53 translation products. Nat Cell Biol 4: 462–467. 12032546 10.1038/ncb801

[11] Courtois S, Verhaegh G, North S, Luciani MG, Lassus P, et al (2002) DeltaN-p53, a natural isoform of p53 lacking the first transactivation domain, counteracts growth suppression by wild-type p53. Oncogene 21: 6722–6728. 12360399 10.1038/sj.onc.1205874

[12] Marcel V, Perrier S, Aoubala M, Ageorges S, Groves MJ, et al (2010) Delta160p53 is a novel N-terminal p53 isoform encoded by Delta133p53 transcript. FEBS Lett 584: 4463–4468. 10.1016/j.febslet.2010.10.005 20937277

[13] Fujita K, Mondal AM, Horikawa I, Nguyen GH, Kumamoto K, et al (2009) p53 isoforms Delta133p53 and p53beta are endogenous regulators of replicative cellular senescence. Nat Cell Biol 11: 1135–1142. 10.1038/ncb1928 19701195 PMC2802853

[14] Nutthasirikul N, Limpaiboon T, Leelayuwat C, Patrakitkomjorn S, Jearanaikoon P (2013) Ratio disruption of the 133p53 and TAp53 isoform equilibrium correlates with poor clinical outcome in intrahepatic cholangiocarcinoma. Int J Oncol 42: 1181–1188. 10.3892/ijo.2013.1818 23404110

[15] Mondal AM, Horikawa I, Pine SR, Fujita K, Morgan KM, et al (2013) p53 isoforms regulate aging- and tumor-associated replicative senescence in T lymphocytes. J Clin Invest 123: 5247–5257. 10.1172/JCI70355 24231352 PMC3859419

[16] Aoubala M, Murray-Zmijewski F, Khoury MP, Fernandes K, Perrier S, et al (2011) p53 directly transactivates Delta133p53alpha, regulating cell fate outcome in response to DNA damage. Cell Death Differ 18: 248–258. 10.1038/cdd.2010.91 20689555 PMC3039296

[17] Bernard H, Garmy-Susini B, Ainaoui N, Van Den Berghe L, Peurichard A, et al (2013) The p53 isoform, Delta133p53alpha, stimulates angiogenesis and tumour progression. Oncogene 32: 2150–2160. 10.1038/onc.2012.242 22733133

[18] Wei J, Noto J, Zaika E, Romero-Gallo J, Correa P, et al (2012) Pathogenic bacterium Helicobacter pylori alters the expression profile of p53 protein isoforms and p53 response to cellular stresses. Proc Natl Acad Sci U S A 109: E2543–2550. 22927405 10.1073/pnas.1205664109 PMC3458371

[19] Slatter TL, Ganesan P, Holzhauer C, Mehta R, Rubio C, et al (2010) p53-mediated apoptosis prevents the accumulation of progenitor B cells and B-cell tumors. Cell Death Differ 17: 540–550. 10.1038/cdd.2009.136 19779492

[20] Campbell HG, Mehta R, Neumann AA, Rubio C, Baird M, et al (2013) Activation of p53 following ionizing radiation, but not other stressors, is dependent on the proline-rich domain (PRD). Oncogene 32: 827–836. 10.1038/onc.2012.102 22484427

[21] Slatter TL, Hung N, Campbell H, Rubio C, Mehta R, et al (2011) Hyperproliferation, cancer, and inflammation in mice expressing a Delta133p53-like isoform. Blood 117: 5166–5177. 10.1182/blood-2010-11-321851 21411755

[22] Campbell HG, Slatter TL, Jeffs A, Mehta R, Rubio C, et al (2012) Does Delta133p53 isoform trigger inflammation and autoimmunity? Cell Cycle 11: 446–450. 10.4161/cc.11.3.19054 22262184

[23] Diaz-Ramos A, Roig-Borrellas A, Garcia-Melero A, Lopez-Alemany R (2012) alpha-Enolase, a multifunctional protein: its role on pathophysiological situations. J Biomed Biotechnol 2012: 156795 10.1155/2012/156795 23118496 PMC3479624

[24] Wygrecka M, Marsh LM, Morty RE, Henneke I, Guenther A, et al (2009) Enolase-1 promotes plasminogen-mediated recruitment of monocytes to the acutely inflamed lung. Blood 113: 5588–5598. 10.1182/blood-2008-08-170837 19182206

[25] Bae S, Kim H, Lee N, Won C, Kim HR, et al (2012) alpha-Enolase expressed on the surfaces of monocytes and macrophages induces robust synovial inflammation in rheumatoid arthritis. J Immunol 189: 365–372. 10.4049/jimmunol.1102073 22623332

[26] Hsiao KC, Shih NY, Fang HL, Huang TS, Kuo CC, et al (2013) Surface alpha-enolase promotes extracellular matrix degradation and tumor metastasis and represents a new therapeutic target. PLoS One 8: e69354 10.1371/journal.pone.0069354 23894455 PMC3716638

[27] Capello M, Ferri-Borgogno S, Cappello P, Novelli F (2011) alpha-Enolase: a promising therapeutic and diagnostic tumor target. FEBS J 278: 1064–1074. 10.1111/j.1742-4658.2011.08025.x 21261815

[28] Sawhney S, Stubbs R, Hood K (2009) Reproducibility, sensitivity and compatibility of the ProteoExtract subcellular fractionation kit with saturation labeling of laser microdissected tissues. Proteomics 9: 4087–4092. 10.1002/pmic.200800949 19701917

[29] Shi HJ, Stubbs R, Hood K (2009) Characterization of de novo synthesized proteins released from human colorectal tumour explants. Electrophoresis 30: 2442–2453. 10.1002/elps.200800767 19639566

[30] Kluza J, Jendoubi M, Ballot C, Dammak A, Jonneaux A, et al (2011). Exploiting mitochondrial dysfunction for effective elimination of imatinib-resistant leukemic cells. PLOS ONE 6(7):e21924 10.1371/journal.pone.0021924 21789194 PMC3138741

[31] Komarova EA, Christov K, Faerman AI, Gudkov AV (2000) Different impact of p53 and p21 on the radiation response of mouse tissues. Oncogene 19: 3791–3798. 10949934 10.1038/sj.onc.1203717

[32] Midgley CA, Owens B, Briscoe CV, Thomas DB, Lane DP, et al (1995) Coupling between gamma irradiation, p53 induction and the apoptotic response depends upon cell type in vivo. J Cell Sci 108 (Pt 5): 1843–1848. 7657708 10.1242/jcs.108.5.1843

[33] Han YH, Park WH (2010) MG132, a proteasome inhibitor, decreased the growth of Calu-6 lung cancer cells via apoptosis and GSH depletion. Toxicol in Vitro 24:1237–5. 10.1016/j.tiv.2010.02.005 20149858

[34] Das R, Burke T, Plow EF (2007) Histone H2B as a functionally important plasminogen receptor on macrophages. Blood 110(10):3763–3772. 17690254 10.1182/blood-2007-03-079392 PMC2077321

[35] Pancholi V (2001) Multifunctional alpha-enolase: its role in diseases. Cell Mol Life Sci 58: 902–920. 11497239 10.1007/PL00000910 PMC11337373

[36] Petrak J, Ivanek R, Toman O, Cmejla R, Cmejlova J, et al (2008) Deja vu in proteomics. A hit parade of repeatedly identified differentially expressed proteins. Proteomics 8: 1744–1749. 10.1002/pmic.200700919 18442176

[37] Sedoris KC, Thomas SD, Miller DM (2010) Hypoxia induces differential translation of enolase/MBP-1. BMC Cancer 10: 157 10.1186/1471-2407-10-157 20412594 PMC2873388

[38] Mizukami Y, Iwamatsu A, Aki T, Kimura M, Nakamura K, et al (2004) ERK1/2 regulates intracellular ATP levels through alpha-enolase expression in cardiomyocytes exposed to ischemic hypoxia and reoxygenation. J Biol Chem 279: 50120–50131. 15459207 10.1074/jbc.M402299200

[39] Chang GC, Liu KJ, Hsieh CL, Hu TS, Charoenfuprasert S, et al (2006) Identification of alpha-enolase as an autoantigen in lung cancer: its overexpression is associated with clinical outcomes. Clin Cancer Res 12: 5746–5754. 17020980 10.1158/1078-0432.CCR-06-0324

[40] Katayama M, Nakano H, Ishiuchi A, Wu W, Oshima R, et al (2006) Protein pattern difference in the colon cancer cell lines examined by two-dimensional differential in-gel electrophoresis and mass spectrometry. Surg Today 36: 1085–1093. 17123137 10.1007/s00595-006-3301-y

[41] Subramanian A, Miller DM (2000) Structural analysis of alpha-enolase. Mapping the functional domains involved in down-regulation of the c-myc protooncogene. J Biol Chem 275: 5958–5965. 10681589 10.1074/jbc.275.8.5958

[42] Lung J, Liu KJ, Chang JY, Leu SJ, Shih NY (2010) MBP-1 is efficiently encoded by an alternative transcript of the ENO1 gene but post-translationally regulated by proteasome-dependent protein turnover. FEBS J 277: 4308–4321. 10.1111/j.1742-4658.2010.07819.x 20849415

[43] Terrier O, Bourdon JC, Rosa-Calatrava M (2013) p53 protein isoforms: key regulators in the front line of pathogen infections? PLoS Pathog 9: e1003246 10.1371/journal.ppat.1003246 23592981 PMC3616980

[44] Terrier O, Marcel V, Cartet G, Lane DP, Lina B, et al (2012) Influenza A viruses control expression of proviral human p53 isoforms p53beta and Delta133p53alpha. J Virol 86: 8452–8460. 10.1128/JVI.07143-11 22647703 PMC3421759

[45] Rohaly G, Korf K, Dehde S, Dornreiter I (2010) Simian virus 40 activates ATR-Delta p53 signaling to override cell cycle and DNA replication control. J Virol 84: 10727–10747. 10.1128/JVI.00122-10 20686026 PMC2950571

[46] Dai RM, Chen E, Longo DL, Gorbea CM, Li CC (1998) Involvement of valosin-containing protein, an ATPase Co-purified with IkappaBalpha and 26 S proteasome, in ubiquitin-proteasome-mediated degradation of IkappaBalpha. J Biol Chem 273: 3562–3573. 9452483 10.1074/jbc.273.6.3562

[47] Chang JW, Koike T, Iwashima M (2009) hnRNP-K is a nuclear target of TCR-activated ERK and required for T-cell late activation. Int Immunol 21: 1351–1361. 10.1093/intimm/dxp106 19880579 PMC2779832

[48] Valle CW, Min T, Bodas M, Mazur S, Begum S, et al (2011) Critical role of VCP/p97 in the pathogenesis and progression of non-small cell lung carcinoma. PLoS One 6: e29073 10.1371/journal.pone.0029073 22216170 PMC3245239

[49] Gao R, Yu Y, Inoue A, Widodo N, Kaul SC, et al (2013) Heterogeneous nuclear ribonucleoprotein K (hnRNP-K) promotes tumor metastasis by induction of genes involved in extracellular matrix, cell movement, and angiogenesis. J Biol Chem 288: 15046–15056. 10.1074/jbc.M113.466136 23564449 PMC3663525

[50] Zhou R, Shanas R, Nelson MA, Bhattacharyya A, Shi J (2010) Increased expression of the heterogeneous nuclear ribonucleoprotein K in pancreatic cancer and its association with the mutant p53. Int J Cancer 126: 395–404. 10.1002/ijc.24744 19609950 PMC2795109

[51] Li F, Zhang D, Fujise K (2001) Characterization of fortilin, a novel antiapoptotic protein. J Biol Chem 276: 47542–47549. 11598139 10.1074/jbc.M108954200

[52] Tuynder M, Susini L, Prieur S, Besse S, Fiucci G, et al (2002) Biological models and genes of tumor reversion: cellular reprogramming through tpt1/TCTP and SIAH-1. Proc Natl Acad Sci U S A 99: 14976–14981. 12399545 10.1073/pnas.222470799 PMC137530

[53] Tuynder M, Fiucci G, Prieur S, Lespagnol A, Geant A, et al (2004) Translationally controlled tumor protein is a target of tumor reversion. Proc Natl Acad Sci U S A 101: 15364–15369. 15489264 10.1073/pnas.0406776101 PMC523462

[54] Love IM, Shi D, Grossman SR (2013) p53 Ubiquitination and proteasomal degradation. Methods Mol Biol 962: 63–73. 10.1007/978-1-62703-236-0_5 23150437

[55] Markowitz J, Rustandi RR, Varney KM, Wilder PT, Udan R, et al (2005) Calcium-binding properties of wild-type and EF-hand mutants of S100B in the presence and absence of a peptide derived from the C-terminal negative regulatory domain of p53. Biochemistry 44: 7305–7314. 15882069 10.1021/bi050321t

[56] Trinidad AG, Muller PA, Cuellar J, Klejnot M, Nobis M, et al (2013) Interaction of p53 with the CCT complex promotes protein folding and wild-type p53 activity. Mol Cell 50: 805–817. 10.1016/j.molcel.2013.05.002 23747015 PMC3699784

[57] Yin X, Fontoura BM, Morimoto T, Carroll RB (2003) Cytoplasmic complex of p53 and eEF2. J Cell Physiol 196: 474–482. 12891704 10.1002/jcp.10329

[58] King FW, Wawrzynow A, Hohfeld J, Zylicz M (2001) Co-chaperones Bag-1, Hop and Hsp40 regulate Hsc70 and Hsp90 interactions with wild-type or mutant p53. EMBO J 20: 6297–6305. 11707401 10.1093/emboj/20.22.6297 PMC125724

[59] Zhang Y, Wang JS, Chen LL, Zhang Y, Cheng XK, et al (2004) Repression of hsp90beta gene by p53 in UV irradiation-induced apoptosis of Jurkat cells. J Biol Chem 279: 42545–42551. 15284248 10.1074/jbc.M314213200

[60] Pelisch F, Pozzi B, Risso G, Munoz MJ, Srebrow A (2012) DNA damage-induced heterogeneous nuclear ribonucleoprotein K sumoylation regulates p53 transcriptional activation. J Biol Chem 287: 30789–30799. 10.1074/jbc.M112.390120 22825850 PMC3436322

[61] Moumen A, Magill C, Dry KL, Jackson SP (2013) ATM-dependent phosphorylation of heterogeneous nuclear ribonucleoprotein K promotes p53 transcriptional activation in response to DNA damage. Cell Cycle 12: 698–704. 10.4161/cc.23592 23343766 PMC3594270

[62] Dreesen O, Chojnowski A, Ong PF, Zhao TY, Common JE, et al (2013) Lamin B1 fluctuations have differential effects on cellular proliferation and senescence. J Cell Biol 200: 605–617. 10.1083/jcb.201206121 23439683 PMC3587829

[63] Rho SB, Lee JH, Park MS, Byun HJ, Kang S, et al (2011) Anti-apoptotic protein TCTP controls the stability of the tumor suppressor p53. FEBS Lett 585: 29–35. 10.1016/j.febslet.2010.11.014 21081126

[64] Chen W, Wang H, Tao S, Zheng Y, Wu W, et al (2013) Tumor protein translationally controlled 1 is a p53 target gene that promotes cell survival. Cell Cycle 12: 2321–2328. 10.4161/cc.25404 24067374 PMC3755082

[65] Waterman MJ, Stavridi ES, Waterman JL, Halazonetis TD (1998) ATM-dependent activation of p53 involves dephosphorylation and association with 14–3–3 proteins. Nat Genet 19: 175–178. 9620776 10.1038/542

[66] Stavridi ES, Chehab NH, Malikzay A, Halazonetis TD (2001) Substitutions that compromise the ionizing radiation-induced association of p53 with 14–3–3 proteins also compromise the ability of p53 to induce cell cycle arrest. Cancer Res 61: 7030–7033. 11585729

