# Supplementary figures and images for: Alpha-Enolase Is Upregulated on the Cell Surface and Responds to Plasminogen Activation in Mice Expressing a ∆133p53α Mimic
Source: PLoS One. 2015 Feb 2;10(2):e0116270. doi: 10.1371/journal.pone.0116270 (PMC4313950; doi:10.1371/journal.pone.0116270)

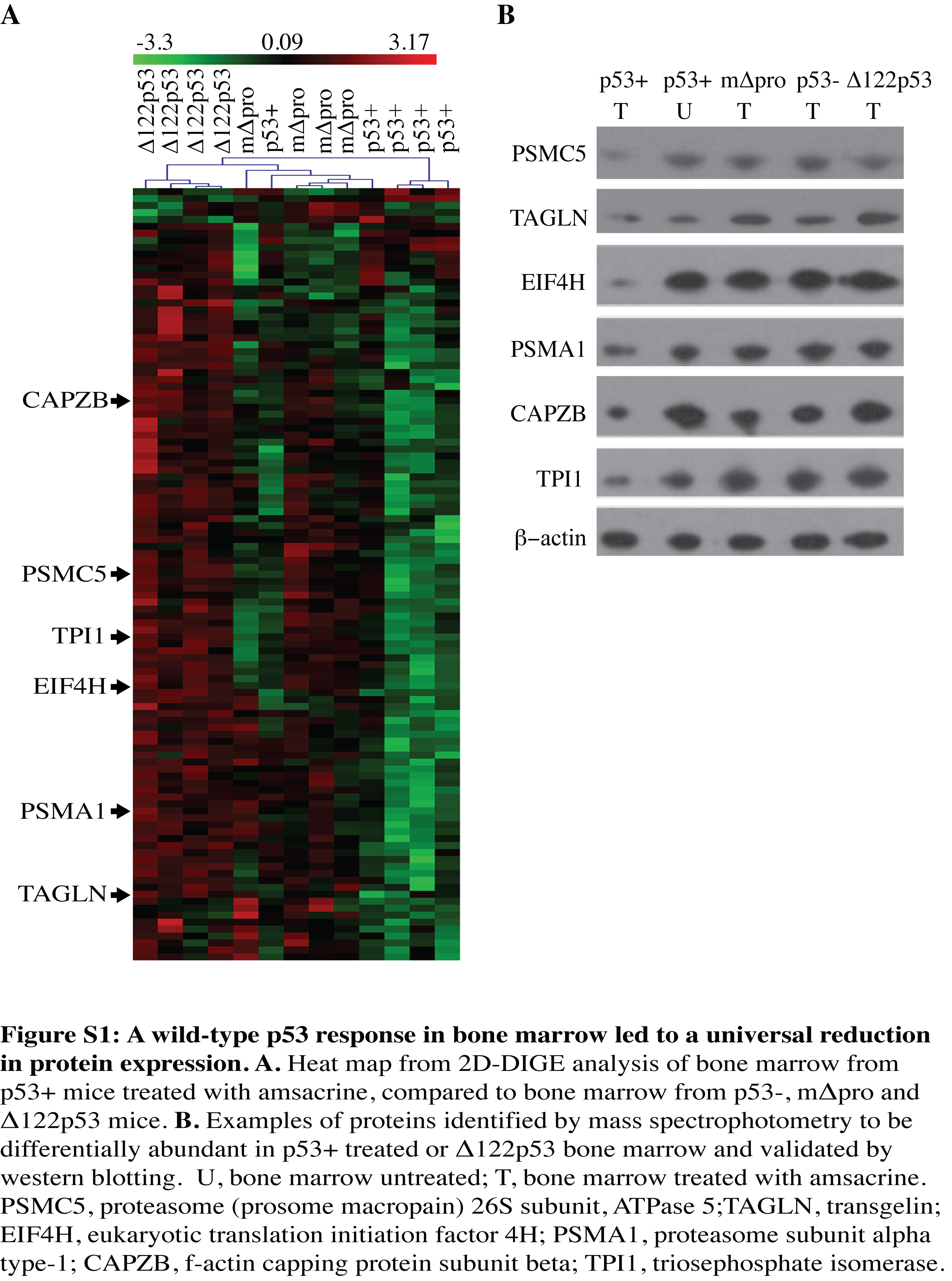

Supplement: S1 Fig — (TIF) [file pone.0116270.s001.tif]

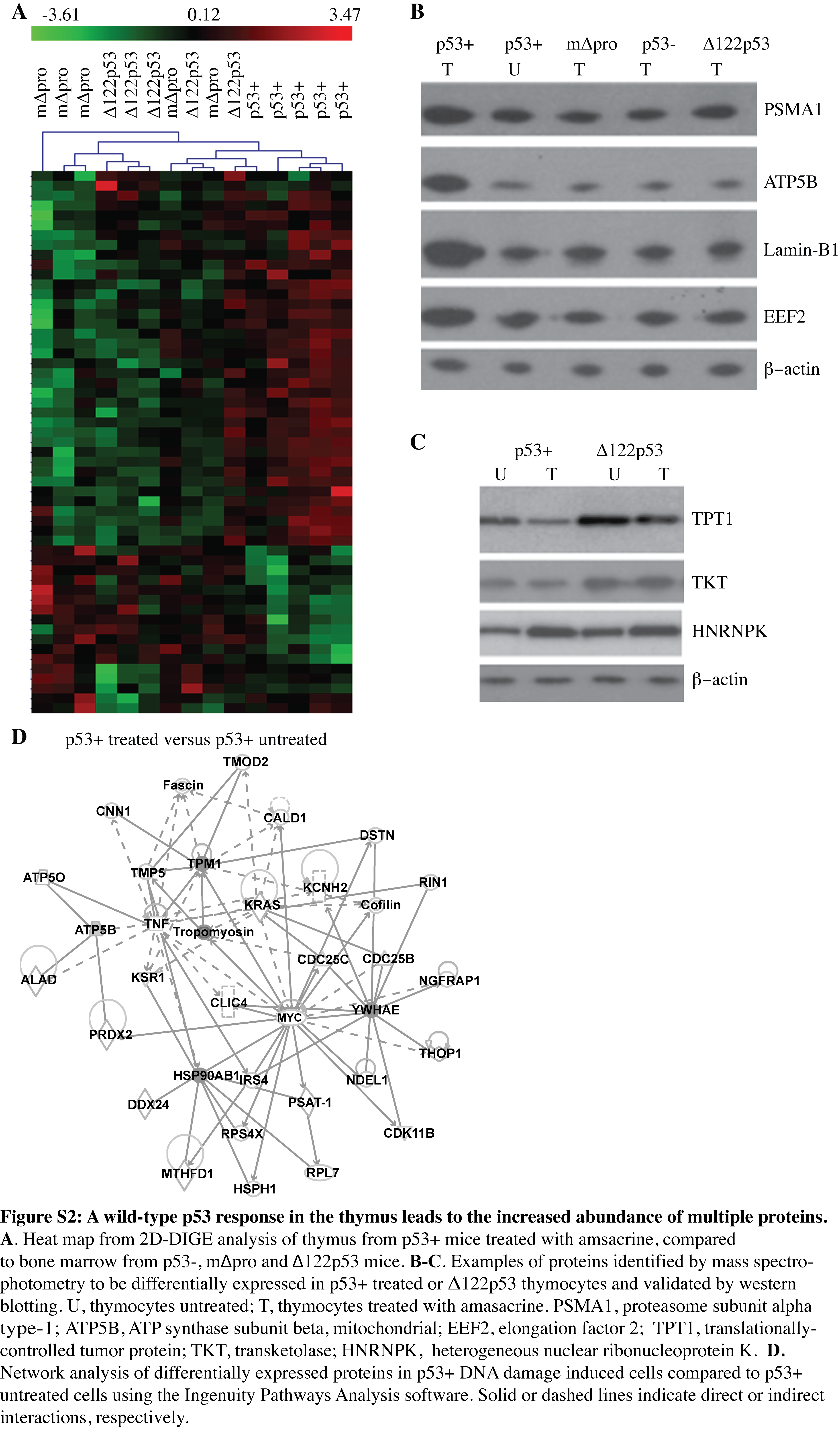

Supplement: S2 Fig — (TIF) [file pone.0116270.s002.tif]

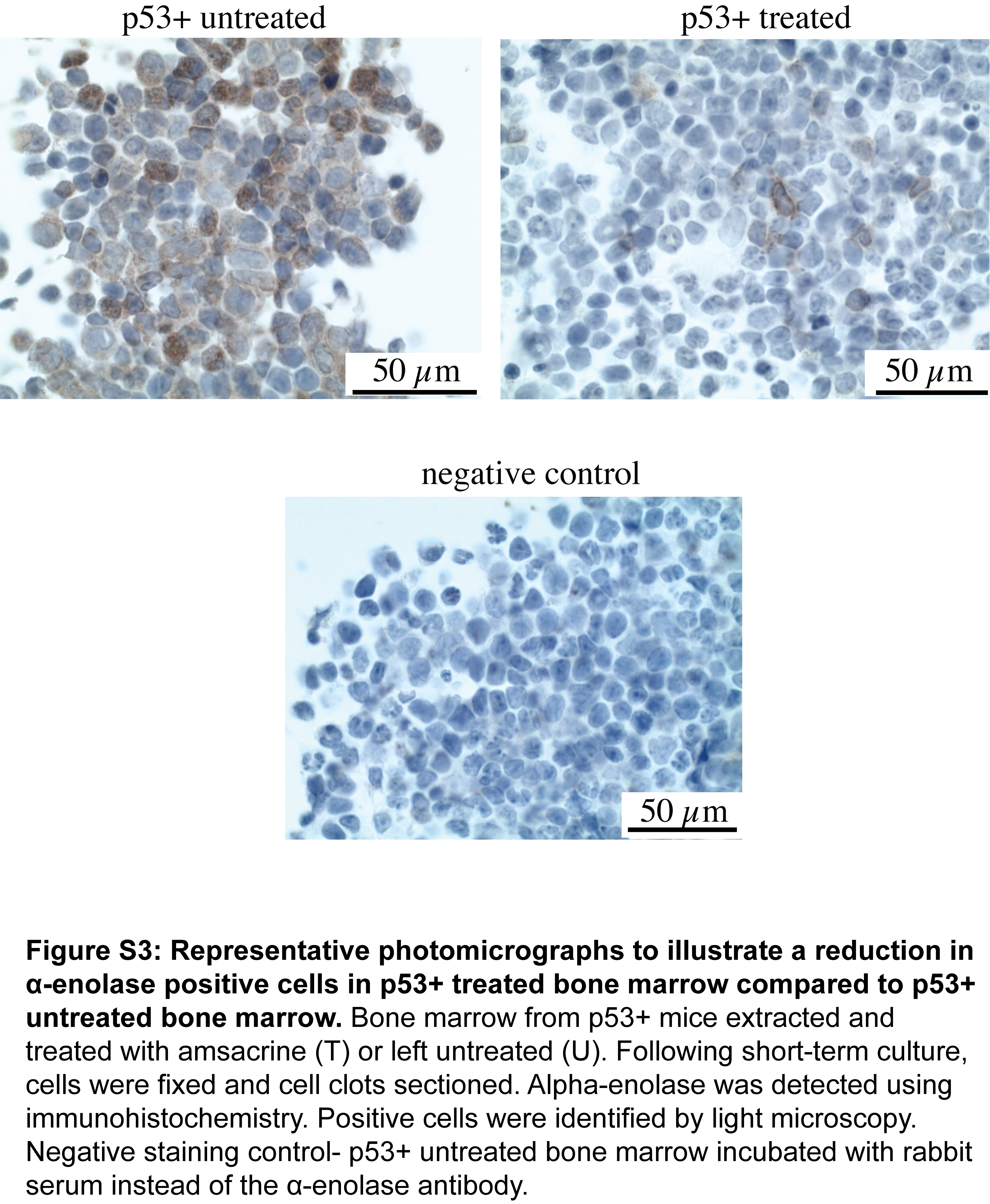

Supplement: S3 Fig — (TIF) [file pone.0116270.s003.tif]
